# Targeting mitochondrial dynamics proteins for the treatment of doxorubicin-induced cardiotoxicity

**DOI:** 10.3389/fmolb.2023.1241225

**Published:** 2023-08-03

**Authors:** Rui Chen, Mengwen Niu, Xin Hu, Yuquan He

**Affiliations:** ^1^ Department of Cardiology, China-Japan Union Hospital of Jilin University, Changchun, China; ^2^ Department of Rheumatology and Immunology, China-Japan Union Hospital of Jilin University, Changchun, China

**Keywords:** doxorubicin (Dox), cardiotoxicity, anticancer effect, mitochondrial dynamics, pharmacological and non-pharmacological interventions

## Abstract

Doxorubicin (DOX) is an extensively used chemotherapeutic agent that can cause severe and frequent cardiotoxicity, which limits its clinical application. Although there have been extensive researches on the cardiotoxicity caused by DOX, there is still a lack of effective treatment. It is necessary to understand the molecular mechanism of DOX-induced cardiotoxicity and search for new therapeutic targets which do not sacrifice their anticancer effects. Mitochondria are considered to be the main target of cardiotoxicity caused by DOX. The imbalance of mitochondrial dynamics characterized by increased mitochondrial fission and inhibited mitochondrial fusion is often reported in DOX-induced cardiotoxicity, which can result in excessive ROS production, energy metabolism disorders, cell apoptosis, and various other problems. Also, mitochondrial dynamics disorder is related to tumorigenesis. Surprisingly, recent studies show that targeting mitochondrial dynamics proteins such as DRP1 and MFN2 can not only defend against DOX-induced cardiotoxicity but also enhance or not impair the anticancer effect. Herein, we summarize mitochondrial dynamics disorder in DOX-induced cardiac injury. Furthermore, we provide an overview of current pharmacological and non-pharmacological interventions targeting proteins involved in mitochondrial dynamics to alleviate cardiac damage caused by DOX.

## 1 Introduction

Due to population growth and improvements in the early identification and treatment of cancers, the number of cancer survivors is increasing, but the survivors often encounter adverse cardiovascular events linked to their cancer therapy or as a result of the worsening of underlying cardiovascular disease (Curigliano et al., 2016), such as arrhythmia, treatment-induced hypertension, thromboembolic ischemia, or heart failure (Narezkina and Nasim, 2019).

It is well established that anthracyclines are usually highly efficacious in the treatment of solid tumors and hematological malignancies but may cause cardiac damage, usually irreversible, which can affect prognosis (Felker et al., 2000; Octavia et al., 2012; Mitry and Edwards, 2016). Doxorubicin (DOX), an antibiotic anthracycline, is frequently used in the treatment of various cancers, including breast cancer, leukemia, and pediatric cancer. There have been documented cases of heart failure that were shown to be closely related to cumulative DOX dosing. When a cumulative lifetime dose of DOX reached 400 mg/m^2^, congestive heart failure was 5% more likely to occur, and heart failure had a 48% probability of occurring at 700 mg/m^2^ (Swain et al., 2003). It is because of its tendency to cause severe cardiotoxicity that the use of DOX in the treatment of cancer is limited.

To date, several processes have been proposed as potential causes of DOX cardiotoxicity, including DNA damage, mitochondrial dysfunction, oxidative stress, and various types of cell death such as apoptosis (Zhang et al., 2012; Kalyanaraman, 2020; Wallace et al., 2020; Wu et al., 2022a). But effective targeted treatments for DOX cardiotoxicity are still insufficient. Given that cardiac mitochondrial damage is predicted to occur within a few hours after DOX administration (Renu et al., 2018), abnormality of mitochondrial morphology and dysfunction are drawing increasing attention. And amounts of studies demonstrate that the imbalance of mitochondrial dynamics tends to enhance mitochondrial fission and weaken mitochondrial fusion underlying DOX treatment in cardiomyocytes and hearts. Now, a thorough report on the role of mitochondrial dynamics proteins in cardiomyocytes countering DOX treatment is showed in this paper, and the pharmacological and non-pharmacological interventions targeting impaired mitochondrial dynamics is presented. This information can aid in the development of additional cardioprotective measures.

## 2 Current mechanisms of DOX-induced cardiotoxicity: oxidative stress and DNA damage

Mitochondria are a major cellular target of DOX. It has been previously demonstrated that mitochondrial damage is strongly linked to DOX-induced cardiotoxicity (Wallace et al., 2020; Schirone et al., 2022). By accumulation in mitochondria of cardiomyocytes, DOX increase ROS production and decrease energy production, causing cell apoptosis (Songbo et al., 2019). Excessive accumulation of intracellular ROS is associated with the modification of mitochondrial dynamics, which play an important role in maintaining mitochondrial function and mediating mitophagy (Songbo et al., 2019). It is widely accepted that the generation of reactive oxygen species (ROS) via redox cycling is a significant source of dose-dependent cardiotoxicity caused by DOX (Varga et al., 2015; Mitry and Edwards, 2016). Anthracyclines can be converted into semiquinone doxorubicin (SQ-DOX), which is catalyzed by nicotinamide adenine dinucleotide phosphate oxidase and nitric oxide synthases in the cytoplasm (Songbo et al., 2019). Due to its unstable structural properties and a high affinity between DOX and cardiolipin at the inner mitochondrial membrane, SQ-DOX can be easily oxidized in the mitochondria, causing the release and accumulation of ROS (Songbo et al., 2019). Excessive ROS production can cause various types of cellular damage and, eventually, cell death. Because the cardiomyocytes have a higher concentration of mitochondria and the heart has lower levels of antioxidant enzymes such as superoxide dismutase (SOD) than other organs, it makes sense that the heart is more susceptible to damage by anthracycline-induced ROS generation (Songbo et al., 2019). Excessive amounts of ROS can cause the opening of mitochondrial permeability transition pores (mPTP) and lead to irreversible loss of mitochondrial membrane potential (MMP), causing mitochondrial damage and cell death (Zorov et al., 2000). Therefore, previous studies initially used antioxidants to combat the cardiotoxicity of anthracyclines. However, the cardioprotective effects of the antioxidants did not result in therapeutic advantages in avoiding or repairing myocardial dysfunction and heart failure in DOX-treated patients (Broeyer et al., 2014; Yeh and Chang, 2016).

Topoisomerase II (Top2), found in eukaryotic cells, is involved in DNA metabolism. This enzyme plays crucial roles in chromosome organization and segregation to support cell growth by unwinding, unknotting, and untangling genetic material by creating momentary double-stranded breaks in DNA (McClendon and Osheroff, 2007). There are two forms of topoisomerase II (Top2): Top2α and Top2β. TOP2α, undetectable in adult cardiomyocytes, is overexpressed in tumor cells, and DOX exerts cytotoxicity in tumor cells by forming Top2α-DOX-DNA complex, responsible for DNA breakage and tumor cell death (Kalyanaraman, 2020). Unexpectedly, TOP2β in adult cardiomyocytes is also a target of DOX, resulting in a Top2β-DOX-DNA ternary complex that causes DNA breakage, cell death and damages in the heart eventually. So, Top2β in cardiomyocytes is also thought to mediate DOX-induced cardiotoxicity (Kalyanaraman, 2020). By the cardiomyocyte-specific deletion of *Top2β* (encoding Top2β), progressive heart failure caused by DOX can be prevented (Zhang et al., 2012). In addition, during DOX exposure, iron metabolism may be directly disrupted by the interruption of key iron-binding and transporter proteins, resulting in iron accumulation in the mitochondria and ferroptosis, which is a type of iron-dependent programmed cell death (Huang et al., 2022). Several previous studies have demonstrated that DOX-induced mitochondrial iron overload and subsequent mitochondria-dependent ferroptosis are major contributing factors in the cardiotoxicity of anthracyclines (Quagliariello et al., 2021; Shan et al., 2021; Huang et al., 2022). Further investigation is warranted to investigate whether ferroptosis inhibitors can be used as cardiac protectors against anthracycline cardiotoxicity. At present, dexrazoxane (DEX) is the only drug recognized by the FDA as being effective in preventing cardiomyopathy and heart failure brought on by anthracycline anticancer drugs (Zamorano et al., 2017). DEX prevents cardiac Top2β from binding to DOX, thus avoiding the formation of DNA double-strand breaks that can result from anthracycline-TOP2β-DNA cleavage complex (Deng et al., 2014; Bures et al., 2017; Corremans et al., 2019; Hasinoff et al., 2020). Also, DEX acts as an iron chelator, alleviating iron accumulation in mitochondria and subsequent ferroptosis (Xu et al., 2005; Hasinoff et al., 2020). However, it can unexpectedly bind to Top2α, which can result in a disruption of the form of Top2α-DOX-DNA, and thus DEX can reduce the anticancer effect of DOX and even increase the risk of secondary malignancies (Tebbi et al., 2007; Vejpongsa and Yeh, 2014).

Recent studies have demonstrated increased mitochondrial fission and inhibited fusion in cardiomyocytes under DOX treatment, resulting in mitochondrial morphological abnormality and dysfunction, accompanied by excessive ROS production, energy metabolism disorders, cell apoptosis, and cardiac dysfunction (Carvalho et al., 2014; Catanzaro et al., 2019; Huang et al., 2022). What’s more, some compounds have been proven to reduce DOX cardiotoxicity by rebalancing mitochondrial fission and fusion (Osataphan et al., 2020; Wu et al., 2022b), implying the regulation of mitochondrial dynamics imbalance may serve as a potent therapeutic strategy for the reduction of DOX-induced cardiotoxicity.

## 3 Mitochondrial dynamics

### 3.1 Mechanics of mitochondrial dynamics

**FIGURE 1:**
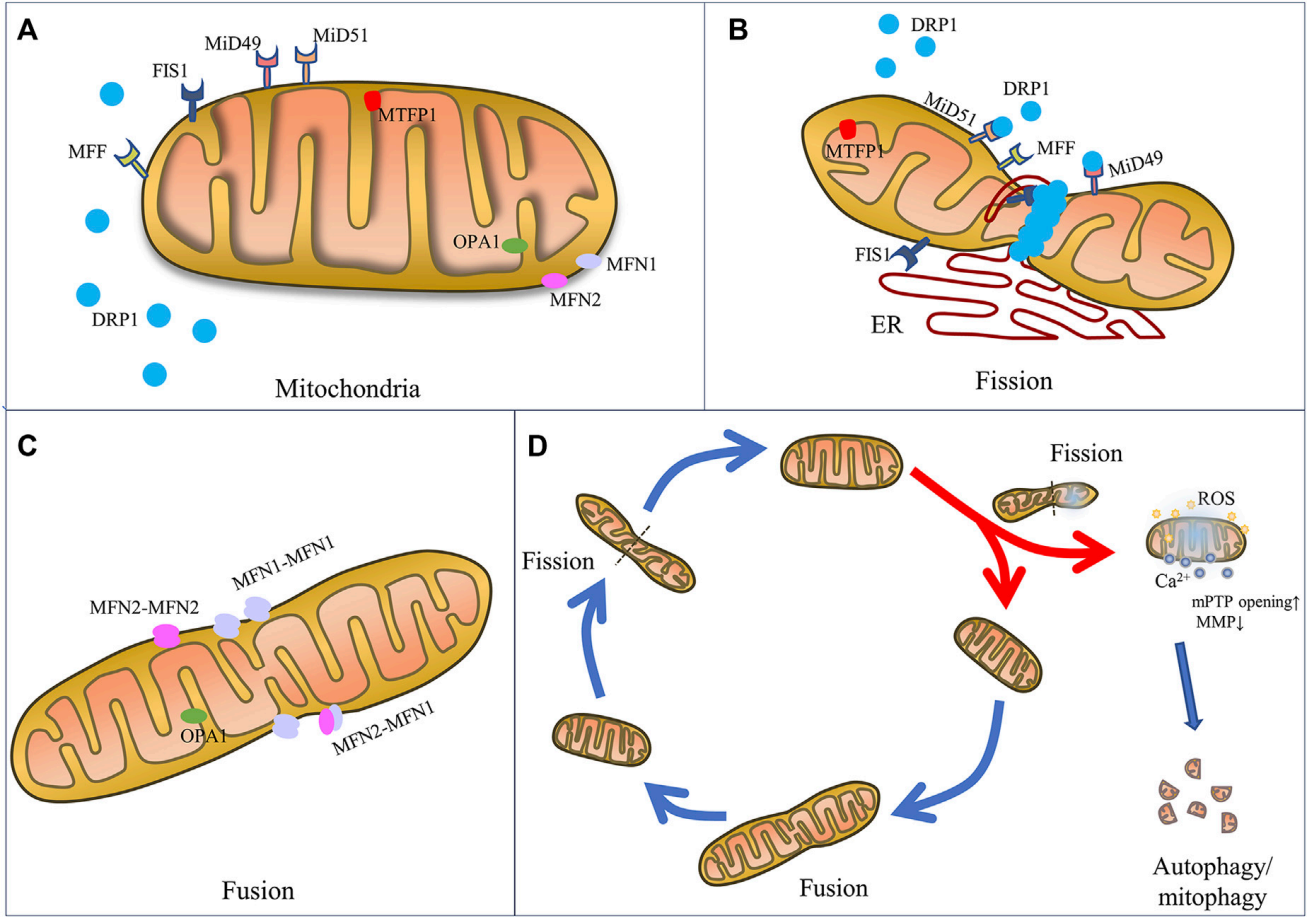
The mechanics of mitochondrial fission and fusion. Schematic representation of mitochondrial fusion and fission machinery and related cell function. **(A)** Mitochondrial dynamics-associated key mediators including pro-fission proteins (DRP1, FIS1, MFF, MiD49, MiD51, and MTF18) and pro-fusion proteins (MFN1, MFN2, and OPA1). **(B)** Mitochondrial fission is mainly regulated by the key player DRP1, OMM receptors (MFF, MiD49, MiD51 and FIS1), which are responsible for the recruitment of DRP1 from the cytosol to mitochondria. And ER wraps mitochondria and identifies the fission site, where DRP1 oligomerizes and induces mem-brane constriction. **(C)** Mitochondrial fusion relies on MFN1 and MFN2 located on the OMM to tether the OMMs of mitochondria, and on OPA1 to mediate fusion of IMM. **(D)** Mitochondrial fate is regulated by fission and fusion to adapt mitochondrial morphology to different stresses. Fission constricts and severs one mitochondrion to daughter mitochondria for cell division or fragmented mitochondria for removal by mitophagy and renewal by fusion into an elongated organelle.

Composed of an inner membrane (IMM) and an outer membrane (OMM) which constantly undergo fusion and fission process, mitochondrial dynamics are finely regulated by a class of dynamin superfamilies known as big GTPases, which are recognized for their capacity to self-assemble and hydrolyze GTP and for their intracellular membrane remodeling activities (Antonny et al., 2016). For example, mitochondrial fission is mainly mediated by a 100 kDa GTPase called dynamin-related protein 1 (DRP1) (Antonny et al., 2016), while mitochondrial fusion is regulated by several specific GTPases, including mitofusin1 (MFN1), mitofusin2 (MFN2) and optic atrophy 1 (OPA1) (Chan, 2020; Giacomello et al., 2020) (Figure 1).

Specifically, mitochondrial fission is the constriction and severance of one mitochondrion to produce fragmented organelles, while mitochondrial fusion is the merging of two or more mitochondria, creating an elongated organelle. The function of mitochondrial fission is to produce two or more daughter mitochondria or mitochondrial fragmentations, which can often be distinguished by varying membrane potentials (Tilokani et al., 2018). In fact, during the mitochondrial life cycle, mitochondrial biogenesis is triggered, causing the formation of new mitochondria, and the mitophagy process occurs, causing the removal of dysfunctional mitochondria or mitochondrial fragmentations generated by mitochondrial fission (Kleele et al., 2021). Fusion is the process by which mitochondria merge to generate a larger organelle, with the fused mitochondria sharing matrix components such as mitochondrial DNA (mtDNA), lipids, proteins, metabolites, and ions (Tilokani et al., 2018). The distribution and integrity of the mitochondria are preserved by these dynamic morphological changes, thereby maintaining function in order to produce sufficient energy and coordinate cellular biological processes, such as calcium homeostasis, redox equilibrium, and apoptosis, in order to achieve cell homeostasis during physiological changes and resist metabolic and environmental stresses (Giacomello et al., 2020).

#### 3.1.1 Mitochondrial fission

The mitochondrial fission process is regulated by various fission factors, including DRP1, mitochondrial fission 1 protein (FIS1), mitochondrial fission factor (MFF), and mitochondrial dynamics proteins 49 kDa and 51 kDa (known as MiD49 and MiD51). In fact, when DRP1 is recruited into the OMM, endoplasmic reticulum (ER) moves toward the mitochondrial periphery and encircles the mitochondria, causing them to tighten (Kraus and Ryan, 2017). In this stage, the diameter of the mitochondrion is decreased from 300–500 nm–150 nm, which causes the development of DRP1–oligo rings (Kamerkar et al., 2018).

As the main mediator of mitochondrial fission, the DRP1 protein has a GTPase effector domain (GED) that is necessary for the conformational changes in DRP1 helices and their constrictive functions in the OMM (Frohlich et al., 2013). Located in cytosol, DRP1 is recruited by receptors to the OMM and induces membrane constriction through oligomerization, forming a ring structure. Because DRP1 lacks a domain that directly binds to phospholipids in the membrane, its recruitment to the outer membrane at sites of contact with ER requires mitochondrial receptors, including the MID49, MID51, and MFF (Loson et al., 2013; Kalia et al., 2018) receptors. When mitochondrial dysfunction and the intracellular AMP/ATP ratio increase, MiD49 and MiD51 recruit DRP1 to the outer membrane, thus promoting DRP1 oligomerization, and MFF specifically recruits DRP1 oligomers with active forms (Kalia et al., 2018). During fission, the enzyme activity of DRP1 is as important as its expression level. The activity of DRP1 is primarily regulated by serine phosphorylation. Phosphorylation at the serine 616 site enhances the activity of DRP1 to promote mitochondrial fission, while phosphorylation at the serine 637 site decreases DRP1 enzyme activity as well as mitochondrial translocation. The phosphorylation ratio between the serine 616 site and the 637 site determines the activity of DRP1 (Chang and Blackstone, 2007; Cribbs and Strack, 2007; Taguchi et al., 2007). DRP1 activity is also post-translationally modified by S-nitrosylation (Lee and Kim, 2018), SUMOylation (Yamada et al., 2021), O-GlcNAcylation (Park et al., 2021), and ubiquitination (Wang et al., 2011; Jin et al., 2021).

FIS1, on the mitochondrial OMM, mediates the translocation of DRP1 from the cytoplasm to the OMM (Macdonald et al., 2014). DRP1 gathers at the mitochondrial division site, forming a lock-lock structure and is then combined with FIS1 to form a complex. The distance and angle between molecules are changed by hydrolysis of GTP and gradually compressed until the mitochondrion transforms into two separate mitochondria. When mitochondrial division is complete, DRP1 is phosphorylated and exfoliates into the cytoplasm (Macdonald et al., 2014). However, FIS1 has recently been implicated in the induction of mitochondrial fragmentation even in the absence of DRP1, a newly discovered function that prevents the fusion process by inhibiting the GTPase activity of MFN1/2 and OPA1 (Yu et al., 2019). Accordingly, by lowering FIS1’s interaction with MFN1/2 and OPA1, the overexpression of MID49 and MID51 has been demonstrated to encourage fission in a DRP1-independent manner (Yu et al., 2021).

In addition, a novel mitochondrial inner membrane protein, mitochondrial fission protein 1 (MTFP1), also known as MTP18, has been discovered to be essential for maintaining mitochondrial integrity, and has consequently been implicated in the regulation of mitochondrial fragmentation in cardiac myocytes and cancer cells (Tondera et al., 2005; Aung et al., 2017). According to Tondera, when MTP18 was overexpressed in COS-7 cells transfected with MTP18-myc expression construct, the shape of the mitochondria changed from filamentous to punctate structures, indicating excessive mitochondrial fragmentation (Tondera et al., 2005). Additionally, RNA interference induced loss of endogenous MTP18 function leads to an increase in fused mitochondria. Furthermore, MTP18 appears to be necessary for mitochondrial fission as it is prevented in cells that have had MTP18 knocked down by RNA interference despite the overexpression of FIS1 (Tondera et al., 2005). And in HL-1 cardiac myocytes, the MTP18 expression was upregulated upon DOX treatment, according to Aung. But knockdown of MTP18 prevented cardiac myocytes from excessive mitochondrial fission by alleviating the DRP1 translocation to the mitochondria and accumulation in the mitochondria, which improved cell apoptosis (Aung et al., 2017). Consistent with the above, it was also found an aberrant overexpression of MTP18 in hepatocellular carcinoma (HCC) cell lines and tumor tissues, and MTP18 promoted tumor growth and metastasis by inducing the progression of cell cycle, epithelial to mesenchymal transition (EMT) and production of MMP9 and suppressing cell apoptosis, which was involved in increased mitochondrial fission and subsequent ROS production (Zhang et al., 2018b).

#### 3.1.2 Mitochondrial fusion

As mitochondria is composed of OMM and IMM, mitochondrial fusion requires two steps. First, the process is initialized by OMM fusion, which is carried out by MFN1 and MFN2. After conformational activation, MFNs from nearby mitochondria oligomerize to form homotypic (MFN1-MFN1 or MFN2-MFN2) or heterotypic (MFN1-MFN2) complexes, which subsequently aid in tethering the OMMs of nearby mitochondria (Rojo et al., 2002; Chen et al., 2003). The GTPase domain on MFN1/2 regulates the outer membranes of the mitochondria to fuse (Chen et al., 2003). MFN1 and MFN2 are highly homologous, but have different hydrolytic activity and fusion efficiency. MFN1 demonstrates stronger hydrolytic activity and pro-fusion efficiency. MFN2 plays a greater role in the formation of the fusion network between mitochondria and ER, as well as in regulating Ca^2+^ homeostasis (Inagaki et al., 2023).

Following OMM fusion, OPA1, located in the IMM, has been proven to be necessary for IMM fusion (Cipolat et al., 2004). In order to mediate the fusion of the IMM, OPA1 creates homotypic (OPA1-OPA1) and heterotypic (OPA1-cardiolipin) complexes after the OMM fuses (Frezza et al., 2006; Yu et al., 2020). Under the control of two mitochondrial metalloproteinases (OMA1 and Yme1L), it can produce short soluble fragments (Wai et al., 2015). OPA1 is divided into at least five fragments, of which the two forms with the highest molecular weight are called L-OPA1, and the other three S-OPA1. In contrast to S-OPA1, which is found in the cristae (pleomorphic invaginations of IMM) of the intermembrane space (IMS), L-OPA1 is fixed on the IMM. Also, OPA1 has been shown to play a role in the modification of the cristae during apoptosis as well as the release of cytochrome c from the cristae (Frezza et al., 2006).

The fission or fusion process is also regulated by interactions between fission proteins and fusion proteins. For example, without DRP1, FIS1 hinders fusion by directly controlling the GTPase activity of MFN1/2 and OPA1 and reducing their interaction with FIS1. Also, it has been demonstrated that overexpression of MID49 and MID51 increases fusion in a DRP1-independent way (Yu et al., 2019; Yu et al., 2021). Therefore, further research is merited in order to determine if fusion and fission function separately and in opposition to each other or whether they work in harmony to modify mitochondrial morphology.

### 3.2 Mitochondrial dynamics and cell function

Changes in mitochondrial morphology are related to key cellular functions, including ROS and Ca^2+^ signaling, energy metabolism, apoptosis, autophagy/mitophagy, inflammation, senescence, and various others (Lee et al., 2004; Yu et al., 2006; Hom et al., 2010; Giacomello et al., 2020). Here, energy metabolism, mitophagy, and cell apoptosis are discussed.

It has been proven that mitochondrial fission is the basis of apoptosis. Pro-apoptotic pore-forming proteins BAX and BAK promote the stabilization of DRP1 at the fission site, leading to mitochondrial fission and network disruption. The above-mentioned changes are accompanied by a transfer of Ca^2+^ signals from the ER to the mitochondria and increased release of cytochrome c, which leads to mitochondrial apoptosis (Wasiak et al., 2007; Prudent et al., 2015). Also, the inhibition of mitochondrial fusion is involved in the process of apoptosis. For example, non-oligomerized MFN1 contributes to promoting BAK oligomerization, which in turn promotes mitochondrial permeability during apoptosis (Ferreira et al., 2019). In addition, OPA1-dependent cristae remodeling is required for the effective release of cytochrome c (Campanella et al., 2008; Landes et al., 2010), and permeability transition pore opening with Ca^2+^ overload in mitochondria contributes to mitochondrial osmotic expansion and cristae dilatation (Bernardi et al., 2015; Prudent et al., 2015).

Mitochondrial dynamics also play an important role in the control of energy metabolism. The IMM is one of the primary sites of oxidative phosphorylation (OXPHOS), and OPA1 overexpression is conducive to the assembly and stabilization of mitochondrial respiratory chain complexes that produce energy (Cogliati et al., 2013). Loss of OPA1 results in mitochondrial malfunction, reduced oxidative phosphorylation and ATP generation, faulty mitochondrial Ca^2+^ signaling, and pro-apoptotic effects (Kushnareva et al., 2013). YME1L and OMA1, specific OPA1-targeting proteases that can cleaved OPA1, increase mitochondrial ATP function leading to enhanced fusion (Mishra and Chan, 2016). And by controlling MFN2 expression, PGC-1 modifies mitochondrial shape, oxygen consumption, and membrane potential, illuminating an intriguing connection between mitochondrial dynamics and biogenesis (Peng et al., 2017). Mitochondrial fission is also related to the energy state of the cell. As a receptor of cell energy, AMPK can directly phosphorylate MFF (one of the OMM receptors of DRP1) during mitochondrial respiratory suppression, promote mitochondrial fission, and then clear damaged mitochondria through autophagy or eventually induce apoptosis (Toyama et al., 2016). PKA also phosphorylates DRP1 at Ser637 during starvation, promoting unwanted mitochondrial fusion, thereby avoiding autophagy and promoting cell survival (Gomes et al., 2011; Rambold et al., 2011).

As we know, mitophagy is an indispensable process, removing damaged or senescent mitochondria in order to maintain mitochondrial function and cell homeostasis, and the fission event is required for mitophagy (Li et al., 2022). Classically, mitophagy is performed in the parkin-dependent mechanism. The phosphatase and tensin homologue (PTEN)-induced kinase 1 (PINK1) accumulates on the OMM and recruits Parkin and promotes its translocation into the mitochondria (Yin et al., 2018). And then, Parkin ubiquitinates several targets such as MFN1, which marks defective mitochondria, and that is a trigger signal for the mitophagy process (Ziviani et al., 2010; Sarraf et al., 2013). Parkin ubiquitinates MFN2, thus affecting ER-mitochondrial binding, as well as mitochondrial dynamics and mitophagy (Sugiura et al., 2013). The resulting action on mitophagy adaptors p62/sequestosome 1/LC3 (microtubule-associated protein 1 light chain 3) facilitates the absorption of damaged mitochondria into autophagosomes and the ensuing autolysosomal digesting (Yamada et al., 2019).

## 4 Mitochondrial dynamics disorder in DOX-induced cardiac injury

Cardiomyocyte, which contains up to 6000 mitochondria and fills 30%–40% of the cell’s volume, is one of the highest ATP-consuming cell types, in which mitochondria are largely found under the serosa, around the nucleus, and between the sarcomeres, allowing them to continually supply energy for cardiac contraction (Cao and Zheng, 2019). Due to their heavy reliance on energy production, cardiomyocytes are particularly susceptible to harmful substances that can affect the mitochondria (Varga et al., 2015). Impairment of mitochondrial dynamics has been observed in many cases of heart disease, including cardiac hypertrophy, heart failure, ischemia/reperfusion (I/R) damage, atherosclerosis (Cao and Zheng, 2019), and metabolic and genetic cardiomyopathies, as well as DOX-induced cardiomyopathy (Osataphan et al., 2020). Under circumstances of stress or disease, cardiac mitochondria undergo structural alterations, mitochondrial dysfunction, cell apoptosis, and various other changes (Li et al., 2020a).

**FIGURE 2:**
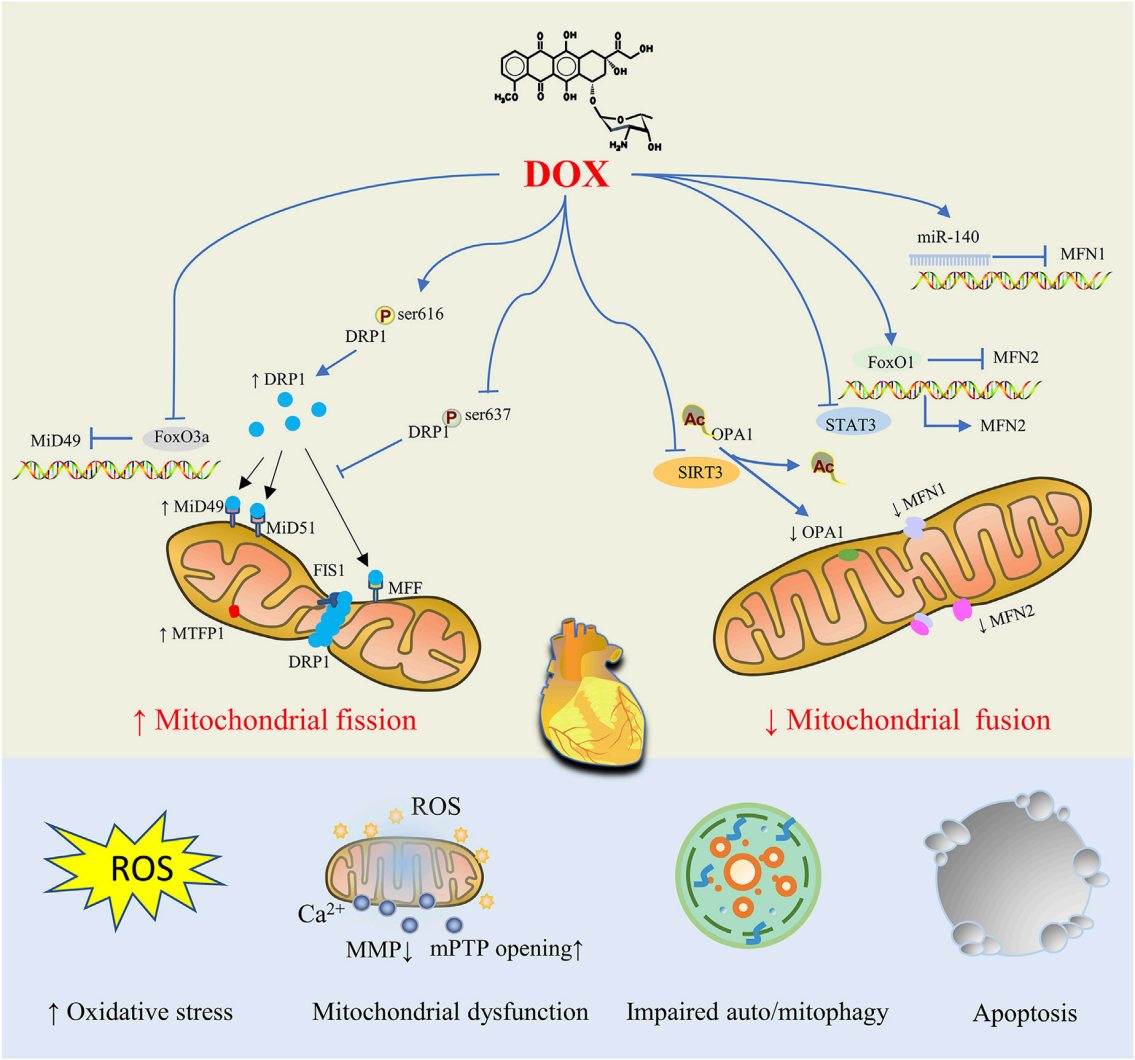
Mitochondrial Dynamics Disorders in doxorubicin (DOX)-induced Cardiac Injury. By controlling proteins involved in mitochondrial fission and fusion, DOX treatment tilts the mitochondrial dynamic balance toward fission, resulting in abnormal mitochondrial structure that ultimately causes oxidative stress, mitochondrial dysfunction, impaired auto/mitophagy, cardiomyocyte apoptosis and cardiac injury.

DOX-stimulated cardiomyocytes display mitochondrial structural abnormalities (such as mitochondrial fragmentation, cristae loss, and matrix destruction), which increase the production of ROS, decrease the MMP, increase the opening of mPTP, disrupt energy metabolism, overburden mitophagy, and cause mitochondrial dysfunction, which results in cardiomyocyte apoptosis (Varga et al., 2015; Wallace et al., 2020). Eventually, dilated cardiomyopathy, heart failure, and ultimately death are possible outcomes of DOX exposure. It is therefore evident that abnormalities in mitochondrial dynamics, characterized by an increase in mitochondrial fission and a decrease in mitochondrial fusion, are the most prominent causes of DOX-induced myocardial injury. For DOX-induced dysregulated mitochondrial dynamics, a number of regulatory mechanics have been proposed, such as changes in the protein expression level of key proteins, post-translational modification and enzyme activity, the process of protein oligomerization, and various possible interactions between these proteins (Figure 2).

### 4.1 Disorders of mitochondrial fission proteins

Previous studies have demonstrated that patients with dilated cardiomyopathy (DCM) have higher levels of DRP1 expression and ser 616 phosphorylation, and that DRP1 may also have a role in mediating mitochondrial fission in Dox-induced cardiotoxicity (Catanzaro et al., 2019). In fact, according to numerous preclinical studies *in vitro* and *in vivo*, DRP1 inhibition had a cardioprotective effect, countering cardiotoxicity caused by DOX. Using siRNA to silence DRP1, mitochondrial fission was inhibited while mitochondrial fusion was increased (Lee et al., 2004). In a DOX-treated H9c2 cardiomyocytes model, excess mitochondrial fission was observed, which was reversed by siRNA-mediated DRP1 knockout (Catanzaro et al., 2019). It was also demonstrated that a DRP1 heterozygous deletion in mice protected against DOX-induced cardiotoxicity, indicating the role of DRP1-dependent mitochondrial dysfunction in DOX-induced cardiotoxicity (Catanzaro et al., 2019). So it was in neonatal rat cardiomyocytes exposed to DOX (Wang et al., 2015).

In response to cellular stress, the phosphorylation of DRP1 at Ser616 increased mitochondrial fission, while the phosphorylation of Ser637 reduced both the enzyme activity of DRP1 and its translocation to mitochondria (Tsushima et al., 2018). Moreover, treatment with DOX in mouse hearts increased Ser616 phosphorylation of DRP1, thus promoting mitochondrial fission (Xia et al., 2017; Miyoshi et al., 2022), which was improved by the administration of LCZ696, a novel angiotensin receptor neprilysin inhibitor (Miyoshi et al., 2022). Also, the inhibition of miR-23a diminished Ser616 phosphorylation of DRP1 and attenuated cardiomyocyte damage caused after DOX administration by directly targeting PGC-1α/p-DRP1 (Du et al., 2019).

It was reported by Aung et al. that MTP18, another mitochondrial fission-associated protein, participated in DOX-induced cardiotoxicity through pro-mitochondrial fission and pro-apoptotic actions (Aung et al., 2017). In HL-1 cardiac myocytes, DOX upregulated the MTP18 expression, and knockdown of MTP18 prevented cardiac myocytes from excessive mitochondrial fission by interfering with the DRP1 translocation to the mitochondria and accumulation in the mitochondria, leading to improved apoptosis (Aung et al., 2017). On the other hand, an inadequate amount of DOX may cause a considerable proportion of cells to suffer mitochondrial fission and death when MTP18 is overexpressed (Aung et al., 2017).

Also, the recruitment of DRP1 to the mitochondrial OMM can be directly mediated by MiD49 (encoded by gene mitochondrial elongation factor 2, MIEF2). Accordingly, knockdown of MIEF2 reduced its association with DRP1, and thus enhanced mitochondrial fusion (Palmer et al., 2011), suggesting that MIEF2 plays a vital role in regulating mitochondrial morphology. Foxo3a, a transcription factor encoded by gene forkhead box O3A, belonging to the Forkhead family, regulates DNA repair, carcinogenesis, cell differentiation, and oxidative stress (Accili and Arden, 2004). When encountering DOX, Foxo3a was downregulated in cardiomyocytes, and cardio-specific Foxo3a transgenic mice showed a reduction of mitochondrial fission, prevention of cardiomyocyte apoptosis, and reversal of cardiac dysfunction according to a study by Luyu Zhou et al. (Zhou et al., 2017). Mechanically, Foxo3a directly targeted MIEF2, and inhibited its expression at the transcriptional and translational levels. MIEF2 and Foxo3a show potential as interesting therapeutic targets that can improve both cancer therapy and cardio-protection.

### 4.2 Disorders of mitochondrial fusion proteins

In addition to the enhancement of mitochondrial fission during DOX treatment, it has been observed that mitochondrial fusion is inhibited, resulting in cardiotoxicity. And the restoration of mitochondrial fusion proteins and the fusion process may help alleviate DOX-induced mitochondrial dysfunction and myocardial injury.

In primary rat cardiomyocytes induced by DOX, it was noted that the expression of MFN2 decreased, leading to decreased fusion and increased ROS generation and cell apoptosis (Tang et al., 2017). In addition, overexpression of MFN2 by transfection of cardiomyocytes with MFN2 CRISPR activation plasmid restored mitochondrial fusion and cell apoptosis (Tang et al., 2017). Furthermore, a recent study conducted by Ding found that MFN2 overexpression promoted mitochondrial fusion and prevented oxidative stress, apoptosis, and cardiac dysfunction caused by DOX (Ding et al., 2022a). DOX exposure led to upregulation of FoxO1 (encoded by gene forkhead box O1), which belongs to the Forkhead family of transcription factors and acts as a key regulator of myocardial homeostasis, thus negatively regulating MFN2 transcription (Ding et al., 2022a). And FoxO1 RNAi dramatically boosted MFN2 mRNA and protein expression, restoring mitochondrial fusion and cell apoptosis (Ding et al., 2022a). Furthermore, treatment with Paeonal, a phenol derived from plants, by activating Stat3, a transcription factor which directly binds to the promoter of MFN2 and upregulates MFN2’s expression at the transcriptional level, enhanced MFN2-mediated mitochondrial fusion, protecting the heart from DOX-induced damage (Ding et al., 2022b).

Several previous studies have demonstrated that miRNA plays a role in regulating key mitochondrial fusion proteins. For example, miR-140 can suppress the expression of MFN1 by targeting the 3′- untranslated region (3′-UTR) and subsequently the knockdown of miR-140 attenuated mitochondrial fission and apoptosis caused by DOX treatment (Li et al., 2014).

It is well known that the cardio-protective inducible enzyme heme oxygenase-1 (HO-1) plays an important role in protecting against DOX induced cardiomyopathy in mice (Hull et al., 2016). While preventing the elevation of the mitochondrial fission mediator Fis1, HO-1 overexpression increased the expression of the fusion mediators MFN1 and MFN2. In summary, the targeting of mitochondrial dynamics proteins was one of the mechanics by which HO-1 played a positive role in cardio-protection against DOX.

In addition to MFN1/2, abnormal expression of OPA1 was also seen in DOX myocardial toxicity. By normalizing expression levels of mitochondrial fusion proteins MFN2, Opa1, and Fis1 (with no change in DRP1 expression), cyclosporine A maintained mitochondrial fusion and preserved mitochondrial function, thereby improving cardiac function and survival in acute and chronic models of cardio-toxicity induced by DOX (Marechal et al., 2011).

And OPA1 was hyperacetylated under stress conditions such as those caused by DOX, and this reduced the GTPase activity of OPA1 and inhibited mitochondrial fusion. A study conducted by Sadhana and co-researchers demonstrated that OPA1 was a direct target of SIRT3, a type of mammalian sirtuin located in mitochondria which deacetylates and modifies the enzymatic activities of several mitochondrial proteins (Bell and Guarente, 2011), and OPA1 can be deacetylated at lysine 926 and 931 residues by SIRT3 (Samant et al., 2014). That is, SIRT3-dependent activation of OPA1 can help protect the mitochondrial network of cardiomyocytes and prevent cell death from DOX.

In summary, numerous studies have demonstrated that exposure to DOX causes the upregulation of mitochondrial fission proteins such as DRP1 and the downregulation of the mitochondrial fusion proteins MFN1, MFN2 and Opa1, resulting in mitochondrial fragmentation and a damaged mitochondrial network. Further understanding of the mechanics of mitochondrial imbalance in DOX-induced cardiotoxicity will help confirm mitochondrial fusion and fission associated proteins as prospective therapeutic targets for reducing DOX-induced cardiotoxicity.

## 5 Mitochondrial dynamics disorder in cancer

Numerous studies have revealed that the balance of mitochondrial dynamics affects cellular biochemical processes like apoptosis and metabolism. During tumorigenesis, mitochondrial dynamic imbalance is also frequently reported (Yin et al., 2022). It has been shown that mitochondrial fission enhancement mediated by upregulation of DRP1 and mitochondrial fusion attenuation mediated by downregulation of MFN2 are associated with multiple cancers and promote tumor cell metastasis and drug tolerance, thus tumor progression (Ma et al., 2020; Ding et al., 2022a). Compared with non-metastatic breast cancer cells, metastatic breast cancer cells have higher expression of DRP1 and lower expression of MFN1 (Zhao et al., 2013). Overexpression of Mfn-2, inhibition of Drp-1, or knockdown of Drp-1 can reduce the proliferation of cancer cells and induce increased apoptosis of tumor cells, thereby shrinking tumors in lung, breast, and colon cancer (Inoue-Yamauchi and Oda, 2012; Rehman et al., 2012; Zhao et al., 2013). The DRP1 inhibitor Mdivi-1 chemically sensitizes tumor cells to the cytotoxic effects of chemotherapeutic drugs by inhibiting mitochondrial fission, leading to the accumulation of defective mitochondria, and ultimately causing dose-dependent death of tumor cells (Courtois et al., 2021).

**FIGURE 3:**
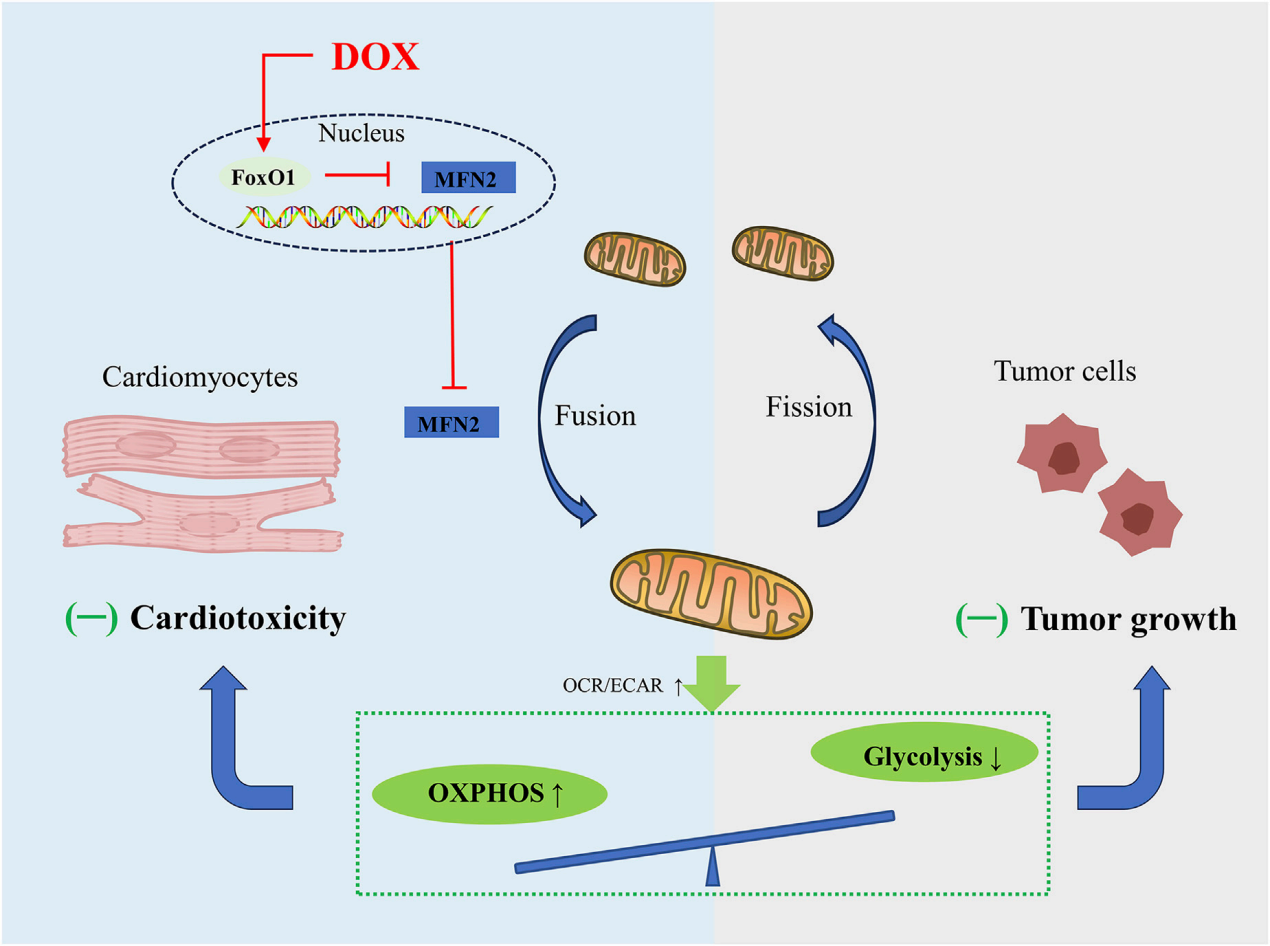
Schematic figure showing that promoting MFN2-mediated mitochondrial fusion induces a similar metabolic transition from glycolysis to OXPHOS in cardiomyocytes and tumor cells, alleviating DOX-induced cardiotoxicity while increasing tumor cell death.

To date, targeting MFN2-mediated mitochondrial fusion has received increasing attention. Mitochondrial dynamics is constantly changing as energy demand. Unlike cardiomyocytes, whose energy is mainly derived from OXPHOS, tumor cells are infinitely proliferating, and even under normal oxygen conditions, their energy is mainly derived from aerobic glycolysis, supplying crucial metabolites for the biosynthetic requirements of prolonged cell growth as well as enough ATP for cancer cells, which is called the Warburg effect (Lunt and Vander Heiden, 2011). Mitochondrial fusion is often associated with the enhancement of OXPHOS in cardiomyocytes (Ding et al., 2022a), while the mitochondrial fission in tumor cells helps to inhibit OXPHOS and enhance aerobic glycolysis (Gao et al., 2020). According to recent research conducted by Ding and co-workers, promoting MFN2 mediated mitochondrial fusion induces a similar metabolic transition from glycolysis to OXPHOS in cardiomyocytes and tumor cells (Ding et al., 2022a), charactered by decrease in the oxygen consumption rate (OCR)-to-extracellular acidification rate (ECAR) ratio. Mechanically, DOX exposure significantly inhibits mitochondrial complex activity and several OXPHOS enzymes, and also inhibits aerobic glycolytic capacity and related glycolytic enzymes in cardiomyocytes and tumor cells, exhibiting greater effect on OXPHOS compared to glycolysis. Due to their different metabolic patterns, enhanced mitochondrial fusion promotes cardiomyocyte survival and induces tumor cell death. Moreover, overexpression of DRP1 promotes mitochondrial fission and significantly weakens the inhibitory effect of MFN2 on DOX-induced cardiac injury (Ding et al., 2022a). In summary, in-depth study of mitochondrial dynamics regulating cell metabolism will provide us with novel therapeutic strategies for alleviating tumor treatment-related cardiotoxicity without sacrificing anticancer effects (Figure 3).

## 6 Modulating the balance of mitochondria dynamics

**FIGURE 4:**
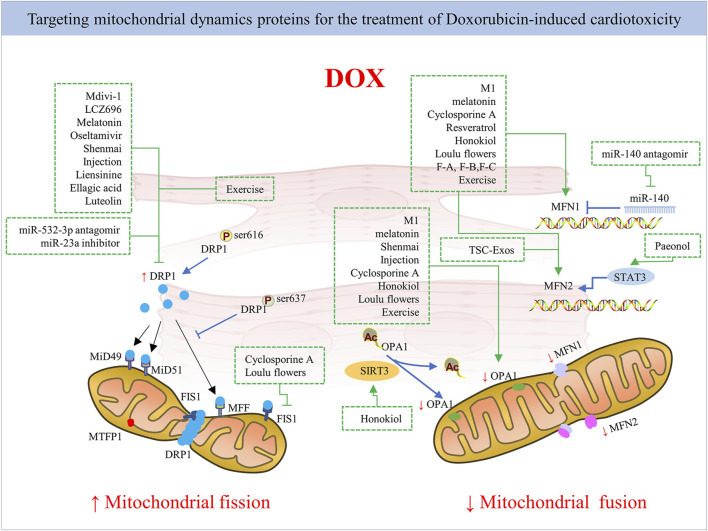
The mechanism of pharmacological strategies and exercise targeting mitochondrial dynamics proteins for DOX-induced cardiotoxicity. Inhibiting mitochondrial fission by targeting mitochondrial fission protein DRP1 mainly or promoting mitochondrial fusion by targeting mitochondrial fusion proteins like MFN1/2 and Opa1 via pharmacological and non-pharmacological strategies exerts cardioprotection against DOX.

Given that DOX impairs mitochondrial dynamics, alleviating mitochondrial fission activation and promoting fusion seem to be effective ways to reduce cardiac function disturbances caused by DOX treatment. Indeed, several studies have confirmed this through gene therapy, pharmacological approaches, and even drug compounds and herbal medicines used in combination in DOX administration (Figure 4). However, there is a need for further research into the application of mitochondrial-targeted agent candidates in clinical DOX cardiotoxicity prevention and treatment without changing the antitumor effect of DOX. Next, we provide an overview of current pharmacological and non-pharmacological interventions targeting mitochondrial dynamics proteins for cardiac damage caused by DOX (Table 1).

**TABLE 1:** An overview of current pharmacological and non-pharmacological interventions targeting mitochondrial dynamics proteins for cardiac damage caused by DOX.

Intervention	Agent	Model	Molecular action in mitochondrial dynamics	Effects on cardiac phenotype	Changes in anticancer effects	Reference
*Pharmaceutical modulators*	Mdivi-1 (1/5/10/20 μM, respectively 30 min before DOX)	H9C2 (5 μM DOX)	↓DRP1 ser 616 phosphorylation	suppress mitochondrial fission and fragmentation	/	Xia et al. (2017)
			↓DRP1 accumulation in mitochondrial	preserve cardiac function		
	M1 (2 mg/kg/day for 30 days) (Mdivi-1, 1.2 mg/kg daily for 30 days)	rat/H9C2 (3 mg/kg DOX on days 0, 4, 8, 15, 22 and 29)	↑expression of MFN1/2 and Opa1	promote mitochondrial fusion	not reduce the anti-cancer efficacy of DOX in MCF7 and MDA-MB231 breast cancer cell lines	Maneechote et al. (2022)
				alleviate mitochondrial fragmentation and apoptosis		
				preserve cardiac function		
*Drug compounds*	LCZ696 (60 mg/kg/day for 4 weeks, beginning on the day after DOX injection)	mice/H9C2 (15 mg/kg, three times/week for 2 weeks in mice)	↓DRP1 ser 616 phosphorylation	alleviate mitochondrial fragmentation and cell apoptosis	/	Xia et al. (2017)
	Metformin (10 mg/ kg/day) and melatonin (250 mg/ kg/day for 30 consecutive days)	rat/H9C2 (3 mg/kg DOX on days 0, 4, 8, 15, 22 and 29)	↓DRP1 ser 616 phosphorylation	improve cardiac mitochondrial dynamic balance, mitophagy and mitochondrial function	synergistic anti-cancer effects with Dox in MCF7 and MDA-MB231	Arinno et al. (2021)
			↑expression of MFN1/2 and OPA1	alleviate cell apoptosis		
	Oseltamivir (OSE) (20 mg/kg for 31 days beginning from 3 days before the first injection of DOX)	rat/H9C2 (15 mg/kg DOX within 2 weeks, 2.5 mg/kg for six times)	↓expression of DRP1	suppress mitochondrial fission and mitophagy	/	Qin et al. (2021)
				preserve cardiac function		
*Natural modulators*	Shenmai Injection (3/1/0.3 g/kg on days 1, 8, 15, 22, and 29)	mice/H9C2 (3 mg/kg DOX on days 9, 16, 23 and 30)	↑DRP1 ser 637 phosphorylation,	improve cardiac mitochondrial dynamic balance	/	Li et al. (2020b)
			↑L-OPA1 to S-OPA1	maintain mitochondrial homeostasis		
	Liensinine (LIEN) (60 mg/kg)	mice/ NMVMs (15 mg/kg for 6 days, single dose in total)	↓DRP1 ser 616 phosphorylation in Rab7/ERK/DRP1 manner	suppress mitochondrial fission	preserve its anti-cancer property of DOX in MDA-MB231 breast cancer cell line	Liang et al. (2020)
				alleviate mitochondrial fragmentation and apoptosis		
				preserve cardiac function		
	Ellagic acid (EA) (1/5/10/20 μM)	NRVMs (10 μM DOX for 18 h)	↓DRP1 ser 616 phosphorylation by inhibition of Bnip3	suppress cell death	not impair the efficacy of DOX in Hela or MCF-7	Dhingra et al. (2017)
	Luteolin (20 µM)	H9c2 and AC16 cells/zebrafish (1 µM Dox for 24 h)	↓DRP1 ser 616 phosphorylation	suppress mitochondrial fission	enhance anti-cancer effects with DOX in 4T1 and MDA-MB-231 and in triple-negative breast cancer of mice models	Shi et al. (2021)
				alleviate apoptosis		
				preserve cardiac function		
	Cyclosporine A (1 mg/kg/48 h)	Mice (a single intraperitoneal bolus for 10 mg/kg follow-up for 1.5 weeks, or 4 mg/kg/week for 5 weeks, follow-up for 16 weeks)	↑expression of MFN2 and Opa1	maintain mitochondrial fusion	/	Marechal et al. (2011)
			↓Fis1	preserve mitochondrial function		
	Resveratrol (RESV) (4 g RESV/kg of diet, equivalent to ∼320 mg RESV/kg/day)	Mice (8 mg/kg/week for 8 weeks)	↑expression of MFN1/2	restore mitochondrial dysfunction	/	Dolinsky et al. (2013)
				preserve cardiac function		
	Honokiol (HKL) (10 μM)	NRVMs (2μM DOX for 24 h)	↑expression of MFN1 and Opa1	promote mitochondrial fusion	not impair the efficacy of DOX in mice implanted with PC3 cells	Pillai et al. (2017)
				restore mitochondrial dysfunction		
	Loulu flowers (LLF) (800 μg/mL in H9C2; pretreated with 100, 200 and 400 μg/mL in zebrafish)	H9C2/zebrafish (0.75 μM DOX for 24 h in H9C2 or zebrafish)	↑OPA1, MFN1	improve cardiac mitochondrial dynamic balance	/	Hu et al. (2022)
			↓MFF and Fis1	maintain mitochondrial homeostasis		
	F-A, F-B, F-C (10 µM)	H9C2 (2.5 µM DOX for 24 h)	↑expression of MFN1/2	maintain the stability of mitochondrial structure and function	/	Zhou et al. (2023)
	Paeonol (Pae) (75, 150, or 300 mg/kg each day after DOX)	rat/NRVMs (5 mg/kg Dox on days 1, 6 and 11, 3 times/2 weeks)	↑expression of MFN2 via the PKCε-Stat3-MFN2 pathway	promote mitochondrial fusion	not interfere with DOX’s antitumor efficacy in B16 melanoma, SNU-368, Hepa 1–6 and 4 T1 cell	Ding et al. (2022b)
				restore mitochondrial dysfunction		
*miRNAs and exosomes*	miR-532-3p antagomir	NRVMs/mice (DOX 4 mg/kg/week for 4 weeks)	↓DRP1 accumulation in mitochondria by targeting ARC	inhibit mitochondrial fission and cell death	not affect DOX-induced apoptosis in cancer cells including Hela, SGC-7901, SW-480 and HepG-2	Wang et al. (2015)
	miR-23a inhibitor	NRVMs (1, 3, or 5 μM DOX for 24 h)	↓DRP1 Ser616 phosphorylation mediated by miR-23a directly targeting PGC-1α/p-DRP1	inhibit mitochondrial fission	/	Du et al. (2019)
				preserve cardiac function		
	miR-140 antagomir	NRVMs/mice (1 μM DOX for 0-15 h)	↑expression of MFN1 through ablating the relationship between 3'-UTR of miR-140 and MFN1	attenuate mitochondrial fission and apoptosis	/	Li et al. (2014)
	TSC-Exos	mice/H9C2 (DOX 5 mg/kg/week for 4 weeks)	↑expression of MFN2	recover mitochondrial fusion,	/	Duan et al. (2023)
				alleviate apoptosis		
*Exercise*	Endurance treadmill training (45 min, 5 days/week 10 min before DOX treatment)	Mice (8 mg/kg/week for 8 weeks, sacrificed on 48 h after the final exercise session)	↑expression of MFN1/2	restore mitochondrial dysfunction	/	Dolinsky et al. (2013)
				preserve cardiac function		
	Endurance treadmill training and voluntary free wheel activity (5 weeks before DOX and during DOX treatment)	Rat (DOX 2 mg/kg/week for 7 weeks)	↑expression of MFN1/2 and Opa1	improve cardiac mitochondrial dynamic balance	/	Marques-Aleixo et al. (2018)
			↓DRP1	regulate mitophagy, alleviate cell apoptosis		
	Endurance treadmill training (60 min/day for 4 weeks after the last DOX treatment)	Mice (5 mg/kg/week for 4 weeks)	↑DRP1	enhance the flux of auto/mitophagy by enhancing mitochondrial fission-prone alterations	/	Lee et al. (2020)
			↓OPA1 and MFN2 along with enhancing the flux of auto/mitophagy	preserve cardiac function		

### 6.1 Pharmacological strategies targeting mitochondrial dynamics disorders

Due to their potential as therapeutic strategies for the targeting of mitochondrial disorders, pharmacological strategies that directly regulate mitochondrial fission and fusion processes are being researched intently. According to Emmanouil (Zacharioudakis and Gavathiotis, 2023), based on advancements in uncovering structure-function correlations, protein-protein interaction of effector proteins and regulation mechanics, and development of pharmacological regulators, targeting fusion and fission associated proteins demonstrate a great therapeutic potential in, for example, cancer, cardiovascular disease, and neurological diseases by reversing the imbalanced mitochondrial dynamics in the onset and progression of these pathologies. Specially, the regulation of protein-protein interactions for oligomers formation of DRP1, OPA1, and MFN1/2, as well as the selective regulation of the effects of the OPA1, MFN1/2, and DRP1 GTPase domains, show great potential for the regulation of mitochondrial fusion and fission.

Mitochondrial Division Inhibitor (Mdivi-1), a quinazolinone derivative, selectively inhibits the main regulator of mitochondrial fission DRP1, making it the most effective inhibitor discovered during the chemical screening of mitochondrial division inhibitors (Cassidy-Stone et al., 2008). According to previous studies, Mdivi-1 reduced mitochondrial fission and can play a crucial role in ameliorating heart failure (Givvimani et al., 2012). Mdivi-1 has been shown to provide cytoprotection in DOX-induced cardiomyopathy in *ex vivo* experiments and in Langendorff perfused heart models by reducing ROS formation, lowering intracellular calcium overload, maintaining mitochondrial function, and avoiding cardiac myocyte hypercontractility (Gharanei et al., 2013). Mdivi-1 not only restored DOX-induced cardiac myocyte depolarization and decreased myocardial infarction size (Gharanei et al., 2013), but also affected the anticancer properties of DOX. A study in a rat model showed that DRP1 and p-DRP1 (Ser616) levels were significantly increased in tissues with mitochondrial damage, while expression levels of MFN1/2 and Opa1 were decreased in DOX-induced myocardial tissues (Xia et al., 2017). By preventing cardiac mitochondrial DRP1 accumulation and cytosolic p-DRP1 (Ser616) activation, Mdivi-1 provided strong protection against excessive mitochondrial fission and fragmentation caused by DOX in an *in vivo* environment, thus reversing changes in mitochondrial morphology and improving mitochondrial function (Xia et al., 2017).

There has been very little research into pharmacological regulators targeting mitochondrial fusion molecules. As a cell-permeable phenylhydrazone compound, M1 was the most effective compound in promoting mitochondrial fusion, according to earlier investigations utilizing different models of cardiac injury. Due to selective upregulation of fusion proteins like MFN1/2 and Opa1 (proteins required for mitochondrial fusion), the protective effects of M1 were demonstrated in models of MPP + or staurosporine-induced cytotoxicity, in diabetic cardiomyopathy hearts, and in cases of cardiac I/R damage (Maneechote et al., 2019). According to another study, M1 reestablished the development of the mitochondrial tubular network via the fragmented mitochondria in MFN1 knockout mice or MFN2 knockout mice (Wang et al., 2012). However, M1 treatment had no effect on the mitochondrial elongation activity of MFN1/2 double KO or Opa1 KO embryonic fibroblasts (MEFs) (Wang et al., 2012). This suggests that basal fusion activity is necessary for M1 to elongate mitochondria, as the formation of the mitochondrial network tubules require interaction with the mitochondrial fusion proteins (MFN1/2 and OPA1).

Treatment with M1 or the combination of Mdivi-1 with M1 may be able to protect the heart from the cardiotoxicity caused by DOX, however this is uncertain. In a study recently performed by Maneechote and his co-researchers, the effects of Mdivi-1, M1, and the combination treatment for cardiomyopathy using DOX-treated rats as a model were examined (Maneechote et al., 2022). The study illustrated for the first time that M1 was potent in preventing the cardiotoxic effects of DOX and encouraging mitochondrial fusion by upregulating MFN1/2 and Opa1. This indicates that Mdivi-1 and M1 may have some protective effects against the cardiotoxicity caused by DOX (Maneechote et al., 2022). The process relies on fission/fusion protein expression. Though reduced cardiomyocyte death and improved contractile properties have been achieved by Mdivi-1 and M1, there was no synergistic advantage from the combination of Mdivi-1 and M1 treatment. The effect of the fusion promoter and the fission inhibitor might coincide at this stage, where mitochondrial fission-mediated fragmentation acts as a downstream natural process. Hence, no great advantages were evident when either medication had already reached its peak effectiveness. It is noteworthy that neither Mdivi-1 nor M1 exhibited any negative effects on DOX’s anticancer activities in breast cancer cell lines. These results can provide some knowledge of the potential therapeutic role of mitochondrial dynamic regulation in DOX-induced cardiotoxicity without sacrificing the effectiveness of the treatment’s anti-cancer properties.

However, it is been found that Mdivi-1 also inhibits the activity of complex I, reducing mitochondrial respiration and increasing the production of ROS (Bordt et al., 2017). This unfavorable target effect may limit the clinical application of Mdivi-1. Also, because of *in silico* screens combined with structure-guided drug generation employing the DRP1 GTPase domain, Drpitor and its optimized variant Drpitor1a were found to be more effective and specific than Mdivi-1 in inhibiting DRP1’s GTPase activity (Wu et al., 2020; Zacharioudakis and Gavathiotis, 2023). Additionally, they improved cardioprotective properties against cardiac I/R injury while demonstrating anticancer actions by lowering cell proliferation and triggering lung cancer cell apoptosis (Wu et al., 2020). P110 is receiving increasing attention as another peptide inhibitor that inhibits the interaction between DRP1 and Fis1. By blocking DRP1/Fis1 connections in models of septic cardiomyopathy both *in vivo* and *in vitro*, P110 can lower septic cardiomyopathy’s morbidity and mortality (Haileselassie et al., 2019). Also, by preventing DRP1 activation and mitochondrial translocation, P110 plays a neuroprotective role (Filichia et al., 2016). Further research is merited to determine whether Drpitor and Drpitor1a, as well as P110, play an important role in DOX-induced cardiac damage.

### 6.2 Drug compounds

In addition to pharmacological regulators, recent research suggests that several drug compounds may attenuate DOX cardiotoxicity by operating on mitochondrial dynamics. These compounds are already registered drugs for the treatment of other diseases, and can easily be repurposed for use in cardio-protection from DOX exposure.

LCZ696, acting as a novel angiotensin receptor neprilysin inhibitor and a standard treatment in patients with heart failure with reduced ejection fraction, can inhibit mitochondrial fission by reducing DRP1 phosphorylation at Ser616 and preventing cell apoptosis from DOX exposure. However, its cardioprotective effect can be reversed by DRP1 overexpression (Xia et al., 2017).

Metformin and melatonin, applied clinically in hypoglycemia and sleep disorder, exerted a protective effect on cardiac function during DOX treatment *in vivo* and vitro by improving mitochondrial dynamics balance through suppressing DRP1 phosphorylation at Ser616 and increasing the expression of MFN1/2 and OPA1, accompanied with improved mitochondrial biogenesis and mitophagy (Arinno et al., 2021).

Known as an antiviral classified as a neuraminidase1 (NEU1) inhibitor, oseltamivir (OSE), preserved cardiac function in models of DOX-induced cardiomyopathy, which was associated with the suppression of DRP1-dependent mitochondrial fission and mitophagy (Qin et al., 2021). Among the four varieties of NEUs (NEU1, NEU2, NEU3, and NEU4), NEU1 is the most abundantly expressed in the heart and is associated with a number of cardiovascular disorders (Glanz et al., 2019; Zhang et al., 2021). For example, NEU1 was shown to be strongly expressed in the hearts of patients with coronary artery disease, and it was also related to cardiac ischemia/reperfusion injury, atherosclerosis formation, and plaque instability, as well as being a key cause of cardiac hypertrophy (Zhang et al., 2018a; Sieve et al., 2018; Heimerl et al., 2020; Chen et al., 2021). In DOX-induced cardiomyopathy in rat models, DRP1 expression was increased by the elevated NEU1, which subsequently boosted mitochondrial fission and PINK1/Parkin pathway-mediated mitophagy. This created a negative feedback loop that led to myocardial apoptosis and cell death (Qin et al., 2021). In summary, the above-mentioned study showed that NEU1 promoted DRP1-dependent mitochondrial fission and mitophagy, which was a critical initiator of DOX-induced cardiomyopathy, but NEU1 inhibition mediated by its inhibitors such as OSE demonstrated novel cardio-protective effects against DOX-induced cardiotoxicity.

### 6.3 Natural regulators

There have also been several studies on natural compounds as potent cardioprotective agents for alleviating DOX-induced cardiotoxicity.

For example, Shenmai Injection suppressed DOX-induced excessive mitochondrial fragmentation in cardiomyocytes by rebalancing mitochondrial dynamics. Mechanically, it reversed a decrease in DRP1 phosphorylation at Ser637 and an increase in DRP1 phosphorylation at Ser616 under treatment with DOX, and simultaneously increased the ratio of L-OPA1 to S-OPA1 via AMPK activation and PI3K/Akt/GSK-3β signaling pathway, which eventually alleviated mitochondria-dependent apoptosis (Li et al., 2020b). Liensinine (LIEN), an isoquinoline alkaloid derived from plants, is a recently discovered mitophagy inhibitor that can work in combination with DOX to treat breast cancer, and it was found that LIEN exhibited a beneficial effect in protecting against DOX-induced cardiomyopathy (Zhou et al., 2015). Liang found that LIEN suppressed DRP1 phosphorylation at the Ser616 site via Rab7 overexpression, thus reversing mitochondrial fragmentation and inhibiting mitochondrial fission–mediated cell death (Liang et al., 2020). Similar to LIEN, ellagic acid (EA), a natural polyphenol compound, can also target mitochondrial fission, attenuating DOX-induced DRP1 Ser616 phosphorylation mediated by the inhibition of Bnip3 (Dhingra et al., 2017). Notably, EA did not reduce DOX’s effectiveness in killing Hela or MCF-7 cancer cells. Along these lines, another study observed that Luteolin (LUT), a naturally occurring flavone enriched in plants, ameliorated DOX-induced toxicity in H9c2 and AC16 cells by alleviating DRP1 expression and Ser616 phosphorylation induced by DOX in H9c2 and AC16 cells (Shi et al., 2021). Also, LUT improved the function of cardiac ventricles in zebrafish under DOX treatment. Furthermore, LUT inhibited proliferation and metastasis in mouse models of triple-negative breast cancer while improving the cytotoxicity (Shi et al., 2021).

Cyclosporine A improved DOX-induced cardiac dysfunction and mortality by normalizing the expression levels of MFN2 and Opa1, sustaining mitochondrial fusion, and preserving mitochondrial function (Marechal et al., 2011). Also, resveratrol (RESV), a naturally occurring polyphenol, was found to increase the expression of mitochondrial fusion proteins MFN1 and MFN2, restore mitochondrial dysfunction, and reduce oxidative stress, thereby protecting against DOX-associated cardiac injury (Dolinsky et al., 2013). It has been proven that SIRT3 defends against cardiotoxicity brought on by doxorubicin. Honokiol (HKL), an activator of SIRT3, was found to promote mitochondrial fusion by maintaining OPA and MFN1 levels, improving mitochondrial function and reducing mitochondrial DNA damage *in vivo* and *in vitro* (Pillai et al., 2017). Also, Loulu flowers (LLF), a member of the Compositae family, have been commonly used in the treatment of cardiovascular illnesses because of their detoxifying effects and capacity to eliminate heat (Hu et al., 2022). A study using H9c2 cells and zebrafish models demonstrated that LLF could alleviate cardiac injury via the blocking of NF-κB signaling and the re-balancing of mitochondrial dynamics by alleviation of the aberrant expression of mitochondrial dynamics-related proteins such as OPA1, MFN1, MFF, and Fis1 (Hu et al., 2022), indicating that LLF can alleviate DOX-induced cardiotoxicity. Wenna and co-workers found that, in order to maintain the stability of mitochondrial structure and function, the protective effect against DOX-induced cardiotoxicity in H9c2 cells of three flavonoids obtained by the separation and purification of sea buckthorn seed residue was partially achieved by increasing protein expression of mitochondrial mitofusins (MFN1, MFN2) (Zhou et al., 2023). Paeonol (Pae), a naturally occurring phenol antioxidant derived from the root bark of Paeonia suffruticosa, was approved as a novel mitochondrial fusion promoter in diabetic cardiomyopathy (Liu et al., 2021). A recent study demonstrated that Pae stimulated mitochondrial fusion and protected the heart from DOX-induced damage via the PKCε-Stat3-MFN2 pathway (Ding et al., 2022b). By targeting PKC and subsequently activating Stat3, which was directly bound to the MFN2 promoter and upregulated its expression at the transcriptional level, Pae enhanced MFN2-mediated mitochondrial fusion (Ding et al., 2022b). Furthermore, Pae did not obstruct Dox’s anticancer activities on a variety of tumor cells.

### 6.4 MicroRNAs (miRNAs) and exosomes

Small non-coding RNAs, known as miRNAs, act as negative regulators of target genes by affecting the stability or translation of mRNA (Valencia-Sanchez et al., 2006), involving several physiological and pathological processes. According to previous studies, cardiac morphogenesis, contraction of the heart muscle, and electrical signal conductivity are all regulated by miRNAs (Thum et al., 2007). The manipulation of miRNAs can be used to develop therapeutic strategies. Given the significance of miRNAs in post-transcriptional regulation, miRNAs also significantly contribute to the cardiotoxicity caused by DOX.

MiR-532-3p was found to regulate mitochondrial fission and apoptosis by targeting ARC, an apoptosis repressor with caspase recruitment domain that acts as an abundantly expressed antiapoptotic protein and affects DRP1 accumulation in mitochondria (An et al., 2009; Wang et al., 2015). More importantly, MiR-532-3p did not affect apoptosis of cancer cells during DOX treatment. And as shown above, miR-23a induced DRP1 Ser616 phosphorylation by directly targeting PGC-1α/p-DRP1 to promote mitochondrial fission, mediating cardiomyocyte damage caused by DOX administration (Du et al., 2019). And MiR-23a inhibitor significantly reduced cardiac damage caused by DOX. Mitochondrial fusion protein can also be regulated by miRNAs. Accordingly, miR-140 can directly suppress the expression of MFN1 by targeting the 3′- UTR without affecting the levels of MFN2, OPA1 or DRP1, and anti-miR-140 can attenuate mitochondrial fission and apoptosis caused by DOX treatment (Li et al., 2014).

Exosomes have established their position as a therapeutic tool in recent years, with an increase in the types of exosomes generated by stem cells being employed in the treatment of heart failure (Roberts and Fisher, 2011; Sun et al., 2021). Exosomes generated from trophoblast stem cells (TSC-Exos) have been shown to guard against the cardiotoxicity caused by DOX (Duan et al., 2023), which has been confirmed both in H9c2 cells and in mice. The study showed that TSC-Exos play a cardio-protective role by recovering mitochondrial fusion through increasing MFN2 expression and alleviating myocardial cell loss.

### 6.5 Exercise

For cancer patients and survivors, exercise training has a cardioprotective effect against DOX-induced cardiotoxicity and has the potential to be a non-pharmacological intervention for the prevention and treatment of chemotherapy-induced cardiotoxicity. Many studies have consistently shown that exercise may lead to the preservation of left ventricular systolic function through a variety of mechanisms, including prevention of DOX accumulation and removal, increased cardiac antioxidant enzyme expression, mitochondrial function, and reduced pro-apoptotic signaling (Scott et al., 2011; Gaytan et al., 2023).

Aerobic training exercise prevents DOX-induced impairments in LV systolic and diastolic function and protects the heart against ROS by enhancing endogenous antioxidant protective machinery such as glutathione peroxidase 1, catalase, and manganese superoxide dismutase in cardiac tissue (Kavazis et al., 2010). Also, voluntary exercise training may provide resistance against the cardiac dysfunction and oxidative damage associated with DOX exposure and induce a significant increase in heat shock protein (HSP72) in the heart (Chicco et al., 2005). Due to the high affinity between DOX and cardiolipin in cardiomyocytes, DOX is more likely to accumulate in mitochondria than in cytoplasm, and studies have shown that DOX has a greater affinity for subsarcolemmal mitochondria compared to intermyofibrillar mitochondria, which difference occurs potentially as a result of greater concentrations of cardiolipin within this mitochondrial fraction (Kavazis et al., 2017). And exercise preconditioning greatly improves subsarcolemmal mitochondria homeostasis by acting on redox balance and iron handling in subsarcolemmal mitochondria upon acute DOX treatment (Montalvo et al., 2023). According to Morton, by boosting the expression of mitochondria-specific ATP-binding cassette (ABC) transporters and lowering the accumulation of mitochondrial DOX, 2 weeks of exercise preconditioning is sufficient to prevent cardiorespiratory failure (Morton et al., 2019). We will next highlight the relevant studies of exercise ameliorating DOX myocardial toxicity through the regulation of mitochondrial dynamics.

In mice given DOX, aerobic exercise training (endurance treadmill training before DOX treatment) enhanced the expression of MFN1/2, as well as the mitochondrial electron transport chain complexes, superoxide dismutase, and cardiac sarcoplasmic/ER calcium-ATPase 2a, and thus protected against DOX-associated cardiac injury (Dolinsky et al., 2013).

Recently, Marques conducted a study showing that no matter when exercise was performed prior to or during sub-chronic DOX treatment, both chronic exercise rat models (endurance treadmill training-TM and voluntary free wheel activity-FW) demonstrated cardio-protection. Mitochondrial dynamics and autophagy/mitophagy were regulated, resulting in decreased cell apoptosis and improved cardiac function (Marques-Aleixo et al., 2018). Specifically, in SD rat models of sub-chronic DOX treatment, administration of DOX caused a decrease in the expression of fusion proteins (MFN1, MFN2 and OPA1), and an increase in DRP1 expression. Augmentation in mPTP opening susceptibility and apoptotic signaling has also been observed, as well as activation of autophagy/mitophagy signaling. All of these effects can be prevented by TM and FW before DOX treatment (Marques-Aleixo et al., 2018).

As mentioned above, before receiving DOX treatment, endurance exercise (EXE) preconditioning can provide cardio-protection, but it is unknown if EXE postconditioning has the same effect. A study from Lee Y et al. has elucidated this subject (Lee et al., 2020). EXE mitigated cardiac tissue damage and prevented DOX-induced apoptosis. Additionally, with increased mitochondrial fission (DRP1) and decreased fusion markers (OPA1 and MFN2), EXE dramatically improved the flux of auto/mitophagy (Lee et al., 2020). Here comes the paradox. The study from Lee Y hinted that EXE interventions promoted mitochondrial autophagy by enhancing fission-prone changes in mitochondrial switching proteins, but another study conducted previously by Marques showed that EXE prevented DOX-induced fission changes and reduced mitochondrial autophagy, thereby providing cardio-protection (Marques-Aleixo et al., 2018). These disparate outcomes might be related to the experimental design. Lee Y began EXE intervention after DOX treatment lasting for 4 weeks, which represented a chronic response in which DOX exposure led to mitophagy delinquency where activation of mitophagy was required by enhancing mitochondrial fission-prone alterations. Marques made rats perform EXE for 5 weeks and then treated them with DOX for 7 weeks together with EXE. The rats were then sacrificed 7 days after the last administration of DOX, which represented an acute response (within 7 days) to DOX exposure, resulting in excessive mitochondrial fission and subsequent autophagy dysfunction.

To sum up, several pharmacological and non-pharmacological interventions such as pharmacological inhibitors or modulators targeting key proteins of mitochondrial dynamics, drug compounds, natural modulators, miRNAs and exosomes mentioned above, and even exercise, have proven that the re-balancing of mitochondrial dynamics by inhibiting mitochondrial fission or promoting mitochondrial fusion, whether specifically or not regulate the enzyme activity, expression level, oligomerization and intermolecular interaction of mitochondrial fission/fusion associated proteins, are effective strategies for DOX-induced cardiotoxicity. 1) Measures such as the development of pharmacological regulators targeting DRP1 and MFN2 like Mdivi-1 and M1 are being taken in order to develop novel therapeutic compounds for DOX-induced cardiotoxicity and even other cardiovascular diseases such as ischemia-reperfusion injury and diabetic cardiomyopathy. And whether Drpitor and Drpitor1a, as well as P110, pharmacological inhibitors by inhibiting DRP1’s GTPase activity and blocking DRP1/Fis1 connections, play an important role in DOX-induced heart damage warrants further investigation. In addition, other regulatory proteins of mitochondrial fusion and fission like Fis1, OPA1, and MFN1, are also worthy of consideration as potential therapeutic targets. However, it is absolutely significant to note that there is still a need for determining the dosage and administration of these modulators. 2) As for drugs with the ability to regulate mitochondrial dynamics, previously used to treat other diseases like LCZ696, repurposing such drugs is an effective strategy to bring them into clinical practice more quickly. 3) Also, many natural compounds have been shown to exert cardioprotective effects through different molecular pathways, modulating abnormal mitochondrial dynamics in cardiomyocytes affected by DOX without weakening, in fact often strengthening, DOX’s anticancer capacity. However, it is worth noting that, due to the multi-target effect of drugs, the mechanism of action and side effects of each compound merit thorough study in the pre-clinical model before clinical studies should be carried out. 4) Furthermore, exosomes secreted by stem cells and miRNAs are increasingly being considered as a therapeutic tool for cardiovascular disease, as they can repair heart damage, and bring about cardiac regeneration through re-balancing impaired mitochondrial dynamics. In order to better understand the upstream and downstream mechanisms underlying protein changes, which facilitate the regulation of mitochondrial dynamic imbalance and mitigate DOX-induced myocardial injury, the effects of miRNA and exosomes on mitochondrial fission proteins and fusion proteins should be thoroughly studied. 5) With regard to exercise, based on the above-mentioned previous studies, whether employed as a therapeutic (postconditioning) or preconditioning approach, EXE is recognized as a vital regulator of mitochondrial turnover that can help maintain positive cellular alterations. Therefore, consistent physical exercise may be reasonably considered to be one of the most important non-drug interventions for DOX-induced cardiotoxicity. Further studies are merited to determine the most efficient, effective, and practical combination of EXE intensity and duration to help cancer patients avoid serious cardiac complications caused by DOX.

Last but not least, it is significant to emphasize that mitochondrial fission is a prerequisite for mitophagy, which is essential for mitochondrial quality control in different heart diseases by eliminating damaged mitochondria (Li et al., 2022). Although compared with mitochondrial fission, mitochondrial fusion has certain protective effects, such as inhibiting the release of cytochrome C and improving mitochondrial metabolism, it prevents the selective elimination of damaged mitochondria by mitophagy (Narendra et al., 2010), that is, the over-enhanced fusion of mitochondria may lead to the accumulation of damaged mitochondria and accelerate the progression of cardiomyopathy. However, in the current studies of cardiotoxicity caused by DOX, there are few studies on the usage of high doses or longer periods of mitochondrial dynamics regulators. Fortunately, the treatment time and pattern of exercise mentioned above provide some clues for the relationship between mitochondrial dynamics and mitophagy. According to Nan, mice treated with high dose show a greater degree of myocardial damage by I/R than control mice, and the short-term administration of Mdivi-1 has proved to be beneficial by limiting ischemic stress to a short period while long-term treatment with Mdivi-1 could be detrimental, which is likely because of excessive inhibition of mitochondrial fission which suppresses mitophagy to result in the accumulation of abnormal mitochondria (Nan et al., 2017). So, in the future, a large number of preclinical studies and clinical studies are needed to emphasize the effects of different dosages and duration of regulators so as to maintain the optimal balance between mitochondrial fission and fusion.

## 7 Conclusion

DOX-induced cardiotoxicity is a complex process involving an imbalance of mitochondrial dynamics, mitochondrial dysfunction, activation of apoptotic pathways in addition to DNA damage and oxidative stress. It has been clearly indicated that mitochondrial dynamics disorder is one of the leading causes of DOX-induced cardiotoxicity. Numerous studies have demonstrated that exposure to DOX causes the tendency of mitochondrial dynamics toward stronger mitochondrial fission and weakened mitochondrial fusion via upregulation of mitochondrial fission proteins such as DRP1 and downregulation of the mitochondrial fusion proteins MFN1, MFN2 and OPA1, resulting in mitochondrial fragmentation and a damaged mitochondrial network. Therefore, it is reasonable to consider the modulation of imbalanced mitochondrial dynamics in cardiomyocytes affected by DOX as a potential target.

And so far, several pharmacological inhibitors targeting key proteins of mitochondrial dynamics, drug compounds, natural modulators, miRNAs and exosomes mentioned above, and even exercise have proven that the re-balancing of mitochondrial dynamics is a potent approach to cardioprotection from DOX exposure by regulating the enzyme activity, expression level, oligomerization and intermolecular interaction of mitochondrial fission/fusion associated proteins.

In summary, rebalance of mitochondrial dynamics proteins is a potential therapeutic strategy for alleviation and avoidance of cardiotoxicity induced by DOX. Specially, inhibiting DRP1-mediated mitochondrial fission and enhancing MFN2-mediated mitochondrial fusion to promote cellular energy metabolic pattern shift may be a very promising therapeutic strategy, as it holds the promise of “killing two birds with one stone”, that is reducing cardiac adverse reactions caused by Dox while improving its anticancer performance. But further research is merited in order to better understand the upstream and downstream molecular mechanics of these proteins, as well as the interactions of mitochondrial dynamics with autophagy and mitophagy in order to safeguard patients from the potentially fatal cardiotoxic effects of DOX.
